# Toward a dual-learning systems model of speech category learning

**DOI:** 10.3389/fpsyg.2014.00825

**Published:** 2014-07-31

**Authors:** Bharath Chandrasekaran, Seth R. Koslov, W. T. Maddox

**Affiliations:** ^1^SoundBrain Lab, Department of Communication Sciences and Disorders, The University of Texas at AustinAustin, TX, USA; ^2^Institute for Mental Health Research, The University of Texas at AustinAustin, TX, USA; ^3^Institute for Neuroscience, The University of Texas at AustinAustin, TX, USA; ^4^Department of Psychology, The University of Texas at AustinAustin, TX, USA

**Keywords:** dual-learning systems, procedural learning, reflective, reflexive, aging, depression, computational modeling

## Abstract

More than two decades of work in vision posits the existence of dual-learning systems of category learning. The *reflective* system uses working memory to develop and test rules for classifying in an explicit fashion, while the *reflexive* system operates by implicitly associating perception with actions that lead to reinforcement. Dual-learning systems models hypothesize that in learning natural categories, learners initially use the reflective system and, with practice, transfer control to the reflexive system. The role of reflective and reflexive systems in auditory category learning and more specifically in speech category learning has not been systematically examined. In this article, we describe a neurobiologically constrained dual-learning systems theoretical framework that is currently being developed in speech category learning and review recent applications of this framework. Using behavioral and computational modeling approaches, we provide evidence that speech category learning is predominantly mediated by the reflexive learning system. In one application, we explore the effects of normal aging on non-speech and speech category learning. Prominently, we find a large age-related deficit in speech learning. The computational modeling suggests that older adults are less likely to transition from simple, reflective, unidimensional rules to more complex, reflexive, multi-dimensional rules. In a second application, we summarize a recent study examining auditory category learning in individuals with elevated depressive symptoms. We find a deficit in reflective-optimal and an enhancement in reflexive-optimal auditory category learning. Interestingly, individuals with elevated depressive symptoms also show an advantage in learning speech categories. We end with a brief summary and description of a number of future directions.

## INTRODUCTION

Fast and accurate categorization is fundamental to the survival of all organisms. The rabbit must categorize a sound as “friend,” “foe,” or a “gust of wind” to determine whether to approach, run, or continue with the current behavior. The Emergency Medical Technician (EMT) must categorize the ausculatory lung sounds heard through a stethoscope as indicative of “fluid” or “no fluid” when determining whether to conduct additional tests or inform the patient that their lungs are clear. The umpire in cricket must decide if a batsman is “out” or “not out” after weighing auditory and visual evidence. These are all categorization problems because there are many information states but only a small number of courses of action.

The psychological study of category learning is long and rich (Bruner et al., 1956; Smith and Medin, 1981; Estes, 1994; Ashby and Maddox, 2005, 2010). Early research focused on single-system models, whereas recent research focuses on multiple-systems approaches. Surprisingly, nearly all of this work focused on the visual domain with little examination of other modalities, including audition. The overriding aim of this paper is to describe a dual-learning systems theoretical framework that is currently being developed in the auditory domain. We attempt to provide a theoretical scaffolding to the emerging field of auditory cognitive science (Holt and Lotto, 2008). In the next sections, we provide a brief history of category learning research starting with single-system approaches and ending with a neurobiologically inspired dual-learning systems approach. We then examine the extent to which the dual-learning systems approach is neurobiologically viable in the auditory domain. Finally, we develop the dual-learning systems framework to speech category learning. Speech category learning involves the mapping of highly variable acoustic cues to perceptual space, akin to a specific type of categorization problem (Holt and Lotto, 2010). However, thus far, speech category learning has been largely viewed as a perceptually encapsulated process. For example, a rich body of literature has examined categorical *perception* (Liberman et al., 1967; Kuhl, 1994, 2004). Categorical perception refers to the percept of invariant categories in sensory events that are discrete and along a continuum. Early studies argued that categorical perception is specific to speech and humans (Liberman et al., 1967). Later studies, however, unequivocally demonstrated that categorical perception extends to other non-speech modalities and exists in non-human species (Kuhl and Miller, 1978; Kuhl, 1985). While the focus on understanding the phenomena of categorical perception still continues (Goldstone and Hendrickson, 2010; Fleming et al., 2013), more recent efforts in the speech sciences have argued the need to study speech perception as a categorization problem (Holt and Lotto, 2010), rather than simply a perceptual problem. In contrast to the auditory domain, a rich prior literature exists in the study of categorization. A goal therefore is to extend the rich theoretical understanding of domain-general learning processes involved in visual category learning literature to speech learning. We conclude with a brief summary and a description of a number of exciting lines of future research.

## SINGLE SYSTEM VS. MULTIPLE SYSTEMS OF CATEGORY LEARNING

Category learning has an extensive history in psychology (Bruner et al., 1956; Smith and Medin, 1981; Nosofsky, 1986b; Estes, 1994; Ashby and Maddox, 2005, 2010). Until the early 1990s, the focus was on developing and testing single-system models of category learning. Three classes of single-system models with multiple instantiations of each were popular during this era: prototype, exemplar, and decision-bound models. Prototype models assume that when asked to assign a stimulus to one of several categories, the participant responds with the category label associated with the most similar prototype (Reed, 1972; Rosch, 1977; Homa et al., 1981; Posner and Petersen, 1990; Smith and Minda, 1998). Exemplar models assume that when asked to assign a stimulus to one of several categories, the participant performs a global match between the representation of the presented stimulus and the memory representation of every exemplar from each contrasting category, selecting the category label associated with the strongest global match (Medin and Schaffer, 1978; Estes, 1986; Hintzman, 1986; Nosofsky, 1986a; Estes, 1994). Decision-bound models assume that the participant learns to assign responses to regions of the perceptual space, and when asked to assign a stimulus to one of several categories, the participant determines into which region the stimulus representation falls and emits the associated response (Ashby and Townsend, 1986; Ashby and Perrin, 1988; Ashby, 1992; Ashby and Maddox, 1993; Maddox and Ashby, 1993). The approach taken by many category learning researchers during this time was to conduct a category learning study and to apply competing models to the data with the aim of identifying the model that provided the best account of the data; the implication being that this “best fitting” model was the correct model (Maddox and Ashby, 1993; McKinley and Nosofsky, 1995; Smith and Minda, 1998). Although a dominant and sometimes fruitful approach, three critical observations cast doubt on this as a viable long-term scientific approach to the study of category learning.

First, research emerged that suggested that many category learning models were mathematically equivalent (Nosofsky, 1990, 1991; Ashby and Maddox, 1993). For example, Ashby and Maddox (1993) (see also Nosofsky, 1990, 1991) showed that prototype, exemplar, and decision-bound models are mathematically equivalent under a broad range of environmental contexts. Thus, in spite of the large differences in psychological processing assumptions across these three classes of models, the models are often equivalent at the level of the data.

**FIGURE 1:**
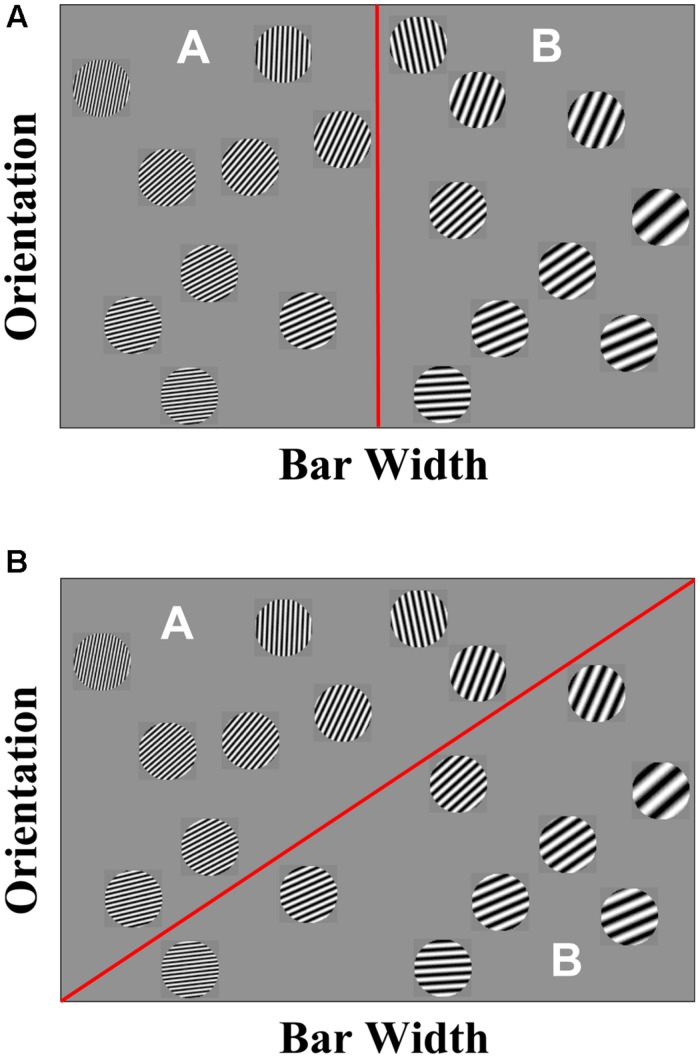
**(A)** Example of a rule-based category learning task in which narrow bar width Gabor patches are in category A and wide bar width Gabor patches are in category B. **(B)** Example of an information-integration category learning task in which no verbalizable rule can be used to describe the strategy that maximizes accuracy.

Second, a number of results suggested that human category learning is mediated by multiple category-learning systems (Nosofsky et al., 1994; Ashby et al., 1998; Erickson and Kruschke, 1998; Reber et al., 2003; Love et al., 2004; Ashby and O’Brien, 2005). One of the strongest pieces of evidence comes from an examination of both of the category structures in **Figure 1**, and the learning profiles associated with each category structure. The stimuli represented in **Figure 1B** were constructed by rotating the items in **Figure 1A** by 45°. Thus, the two spaces are mathematically equivalent and would be learned to equivalent levels by any standard clustering algorithm. Despite this equivalence, humans show very different learning profiles and introspection when asked to solve these tasks. When faced with the task depicted in **Figure 1A**, participants start out near chance and then at some point “get it” and perform nearly optimal. In other words, participants’ learning profile is characterized as a step function. In addition, participants are able to describe the strategy that they used accurately. When faced with the task depicted in **Figure 1B**, participants start out at near chance and then show gradual, incremental learning. Participants are unable to describe the strategy that they used accurately and often say that they went with their “gut” feeling, or “gut reflex.”

This qualitative difference in performance across these structurally equivalent categories led to a number of interesting studies that revealed strong empirical dissociations between the learning of these two category structures. Because single-system models are unable to account simultaneously for more than one or two of these multiple-system results, the field began to question the viability of single-system approaches. Brooks and colleagues suggested one of the earliest multiple-systems approaches, arguing for separate rule-based (RB) and exemplar-based systems (Brooks, 1978; Allen and Brooks, 1991; Regehr and Brooks, 1993). Since then, a number of purely cognitive multiple-systems models have been proposed, with nearly all offering some specific instantiation of Brooks’ RB and exemplar-based systems (Nosofsky et al., 1994; Erickson and Kruschke, 1998; Love et al., 2004).

Finally, a plethora of research examining the neural basis of category learning emerged (Poldrack and Packard, 2003; Nomura et al., 2007). The existence of the neural data weakens the predictive power of the purely cognitive models since they are ambivalent with respect to neuroscience. This revolution opened the door to a number of new methodological approaches.

### A NEUROBIOLOGICALLY BASED DUAL-LEARNING SYSTEMS MODEL (COVIS)

One of the theories of category learning that specifies the constraints imposed by the underlying neurobiology is the COmpetition between Verbal and Implicit Systems (COVIS; Ashby et al., 1998, 2011) model. As we later elaborate, COVIS focuses exclusively on the visual domain. COVIS postulates two learning systems, one reflective and one reflexive^1^. The reflective system is an explicit learning system in the sense that it formulates and tests specific categorization rules using executive attention and working memory. The critical neural structures include prefrontal cortex, anterior cingulate, and anterior caudate nucleus (Lombardi et al., 1999; Monchi et al., 2001; Ashby and Valentin, 2005; Ashby et al., 2005; Filoteo et al., 2005c; Seger and Cincotta, 2006; Nomura et al., 2007; Schnyer et al., 2009). **Figure 1A** displays a simple two-category, RB problem using Gabor patches that vary in spatial frequency and spatial orientation as stimuli.

The strategy that maximizes accuracy is to place low spatial frequency items into category A and high spatial frequency items into category B. This strategy is referred to as an RB or reflective strategy. In contrast, the reflexive system is implicit and procedural and learns to associate stimuli lying in different regions of perceptual space with specific motor outputs as a result of reinforcement via trial feedback. Accurate performance in reflexive categorization requires predecisional integration of stimulus components, and it is therefore often referred to as an information-integration (II) strategy. Learning in this system does not rely on working memory and executive attention, and the critical structures are the posterior caudate, putamen and the supplementary motor area (SMA; Ashby and Waldron, 1999; Maddox and Filoteo, 2001; Poldrack et al., 2001; Aron et al., 2004; Filoteo et al., 2005b; Maddox and Filoteo, 2005; Seger and Cincotta, 2005; Nomura et al., 2007; Seger, 2008; Ashby and Crossley, 2011). **Figure 1B** displays a simple two-category problem. The strategy that maximizes accuracy in **Figure 1B** (unlike the structure in **Figure 1A**) is not easily verbalizable, so an II strategy implemented via the reflexive system is most optimal for categorizing these stimuli.

The COVIS model assumes that the reflective and reflexive learning systems compete throughout category learning. In humans, there appears to be a bias toward reflective dominance. Individuals explicitly test category rules and adjust the weight given to that rule depending on its success or failure. The success or failure of rules is assessed by explicit processing of the feedback. After each trial, utility of a particular rule is updated. Through this method of hypothesis testing, relevant decision bounds are learned. The explicit nature of the reflective system requires use of working memory and executive attention to remember which rules have been used, to process the success or failure of these decision bounds, and to switch between rules. COVIS posits that an accurate reflective system prevents the transfer of control to the striatally mediated reflexive system (Ashby and Maddox, 2010). Learners will therefore continue to use reflective system until the reflexive system is more accurate.

In comparison, during reflexive learning, a striatal unit implicitly associates an abstract cortical–motor response with sensory cells in the sensory association cortex. Learning occurs at cortical–striatal synapses. Such synaptic plasticity is enhanced by a dopamine-mediated reinforcement signal. The timing and nature of feedback in a categorization experiment are crucial to the effectiveness of the reflexive learning system, while working memory is not critical to learning. Despite the different circuitries, both the reflective and reflexive learning systems utilize components within the primary and association sensory regions. For further details, the reader is referred to previous review papers on the COVIS model (Ashby and Maddox, 2010; Ashby et al., 2011). See **Table 1** for a summary of properties of the reflective and reflexive systems.

**Table 1:** Summary of the main properties of the reflective and reflexive systems.

	Learning system	
	Reflective	Reflexive
Description	Explicit and verbalizable	Implicit and non-verbalizable
Neurobiology	Prefrontal cortex; anterior cingulate; head of the caudate nucleus; hippocampus	Putamen, body, and tail of the caudate nucleus; premotor cortex
Mechanism	Operates by formulating and testing categorization rules	Operates by implicitly associating perception with actions that lead to reinforcements.
Working memory/PFC dependence	Dependent on executive attention and working memory	Not dependent on working memory and executive attention; dependent on striatum
Feedback characteristic	Benefits from rich, explicit feedback. Feedback timing not critical	Benefits from minimally informative feedback. Feedback timing is critical

The dual-learning systems approach in general, and COVIS in particular, has gained broad support with evidence from behavioral studies conducted in a variety of areas. These include: healthy adult humans (Ashby and Maddox, 2005, 2010; Grimm and Maddox, 2013; Ashby, 2014; Smith et al., 2014), human children, and older adults (Ridderinkhof et al., 2002; Filoteo and Maddox, 2004; Filoteo et al., 2005a; Racine et al., 2006; Minda et al., 2008; Maddox et al., 2010; Huang-Pollock et al., 2011; Gorlick et al., 2012), non-human animals (Smith et al., 2004, 2010, 2011, 2012a,b), various neuropsychological patient groups (Knowlton and Squire, 1993; Knowlton et al., 1994; Squire and Knowlton, 1995; Knowlton, 1999; Keri, 2003; Filoteo et al., 2005b; Filoteo and Maddox, 2007), as well as using brain imaging techniques such as fMRI (Poldrack et al., 1999, 2001; Cincotta and Seger, 2000, 2007; Poldrack and Packard, 2003; Aron et al., 2004; Poldrack and Rodriguez, 2004; Shohamy et al., 2004; Seger and Cincotta, 2005; Seger and Cincotta, 2006; Nomura et al., 2007; Nomura and Reber, 2008; Seger, 2008; Helie et al., 2010; Seger and Miller, 2010; Waldschmidt and Ashby, 2011) and EEG (Folstein and Van Petten, 2004).

## TOWARD AN AUDITORY VERSION OF COVIS

### NEUROANATOMY

A major focus of this article is to examine the application of the dual-learning systems model to the auditory domain. Previous studies have shown similarities in the organization of the two major sensory domains. In vision, an organizing principle is retinotopy; in audition, topographical organization by frequency (“tonotopy”) has been demonstrated along the auditory pathway. Functionally distinct dorsal and ventral cortical streams are seen both in vision and audition (Romanski et al., 1999; Marois et al., 2000; Rauschecker and Scott, 2009). However, there are some critical differences between the two domains as well. A significant amount of auditory signal processing occurs well before signals reach the auditory midbrain. The visual pathway lacks functional processing centers at the level of the brainstem. The auditory system is subserved by massive efferent (feedback) connectivity that yields substantial top-down control of the lower level auditory centers. In contrast, the efferent connectivity of the visual system is less massive. Functionally, the auditory system is constantly “on” (even when we are asleep) and therefore metabolically more expensive. In monkeys, auditory working memory is less robust and more susceptible to “rewriting” than visual working memory (Scott et al., 2012). In humans, there is a marked difference in recognition memory for visual and auditory objects. The memory for visual images is far greater than for auditory objects (Cohen et al., 2009). Despite these differences, a direct comparison of the two modalities has been challenging due to methodological difficulties in matching the sensory and cognitive load imposed by auditory and visual stimuli. A recent behavioral and computational modeling study matched auditory and visual stimuli on stimulus complexity (static or moving gabor patches vs. moving ripple stimuli) and showed processing similarities between the two modalities in a short-term memory task (Visscher et al., 2007). This study suggests that memory processes are not modality specific. Given inconsistent findings about commonalities/differences between audition and vision, an important question is whether the neural circuitry underlying the dual-learning systems has a parallel in the auditory domain.

**FIGURE 2:**
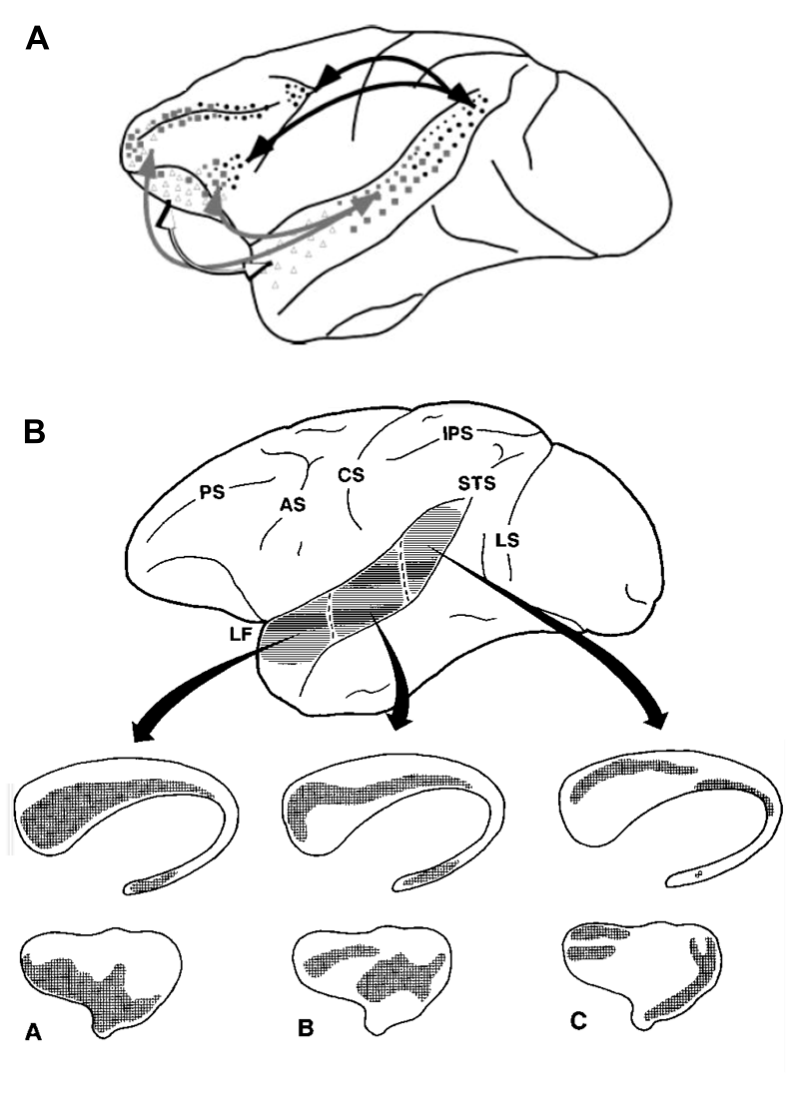
**Neuroanatomy in support of the (A) reflective, and (B) reflexive auditory category learning systems.** Primary and secondary auditory cortices are directly connected to the reflective **(A)** and reflexive **(B)** learning systems. Adapted from Petrides and Pandya (1988) and Yeterian and Pandya (1998).

The bidirectional connectivity among primary, secondary auditory cortices, and the prefrontal cortex is well established (Rauschecker and Scott, 2009). This connectivity forms a clear basis for a functional reflective auditory system. In contrast, relatively little is known about the functional role of the corticostriatal connectivity in audition. In the next few paragraphs, we review the existing work from animal and human models that argue for a reflexive auditory system. Retrograde tracing experiments in animal models show direct connectivity from the auditory thalamus and auditory cortex to the striatum (LeDoux et al., 1991) In cats, auditory cortical projections to the striatum is tonotopic (Reale and Imig, 1983). Retrograde anatomical labeling studies in primates show that the primary and association auditory cortices are bi-directionally connected to the dorsolateral prefrontal cortex and form many-to-one projections to the striatum (Petrides and Pandya, 1988; Yeterian and Pandya, 1998; **Figure 2**).

The connections from the primary auditory cortex to the striatum are relatively sparse. In contrast, connections from the belt region, which surrounds the primary auditory cortex, to the caudate and putamen are more dense (Yeterian and Pandya, 1998). Examining responsivity in the striatum to auditory stimulation using c-fos induction, Arnauld et al. (1996) showed dense Fos-IR within the caudal striatum, and relatively sparse labeling in the rostral striatum. This is in contrast to visual stimulation, which resulted in Fos-IR within the rostral striatum (Arnauld et al., 1996). Despite retrograde labeling studies showing diffuse corticostriatal connectivity patterns, the projections from the auditory system largely converge on to the caudal portion of the striatum (Arnauld et al., 1996). While the previous studies have all examined the corticostriatal projection, there is some evidence for a backprojection from the striatum to the auditory cortex via the pallidum. The functional role of this backprojection is unclear (Parent and Hazrati, 1995). From a functional perspective, a recent study showed that decisions on auditory stimuli are functionally determined by corticostriatal connections in rats. Optogenetic stimulation of the corticostriatal neurons biased the animal’s choice (Znamenskiy and Zador, 2013). In humans, a resting-state connectivity study demonstrated functional connectivity between the putamen and the auditory association area. Connectivity is more robust between the auditory cortex and the putamen relative to the caudate (Di Martino et al., 2008).

Despite the fundamental differences between auditory and visual perception, the brain regions associated with auditory processing are interconnected with the brain regions associated with reflective and reflexive category learning. This connectivity is a good indication that the neurobiology associated with the COVIS model is plausible in both the auditory and visual domains. We next need to determine whether processing in these auditory analogs of reflective and reflexive category learning systems behave in a manner similar to those associated with reflective and reflexive visual category learning. Ultimately, we should approach this with all of the same tools that have been used in the visual domain. This includes behavioral dissociation studies, lifespan research, brain imaging techniques (fMRI, EEG), and neuropsychological patient groups. Our group has made headway using some of these approaches and that work will be reviewed here.

### REFLECTIVE AND REFLEXIVE AUDITORY LEARNING SYSTEMS

**FIGURE 3:**
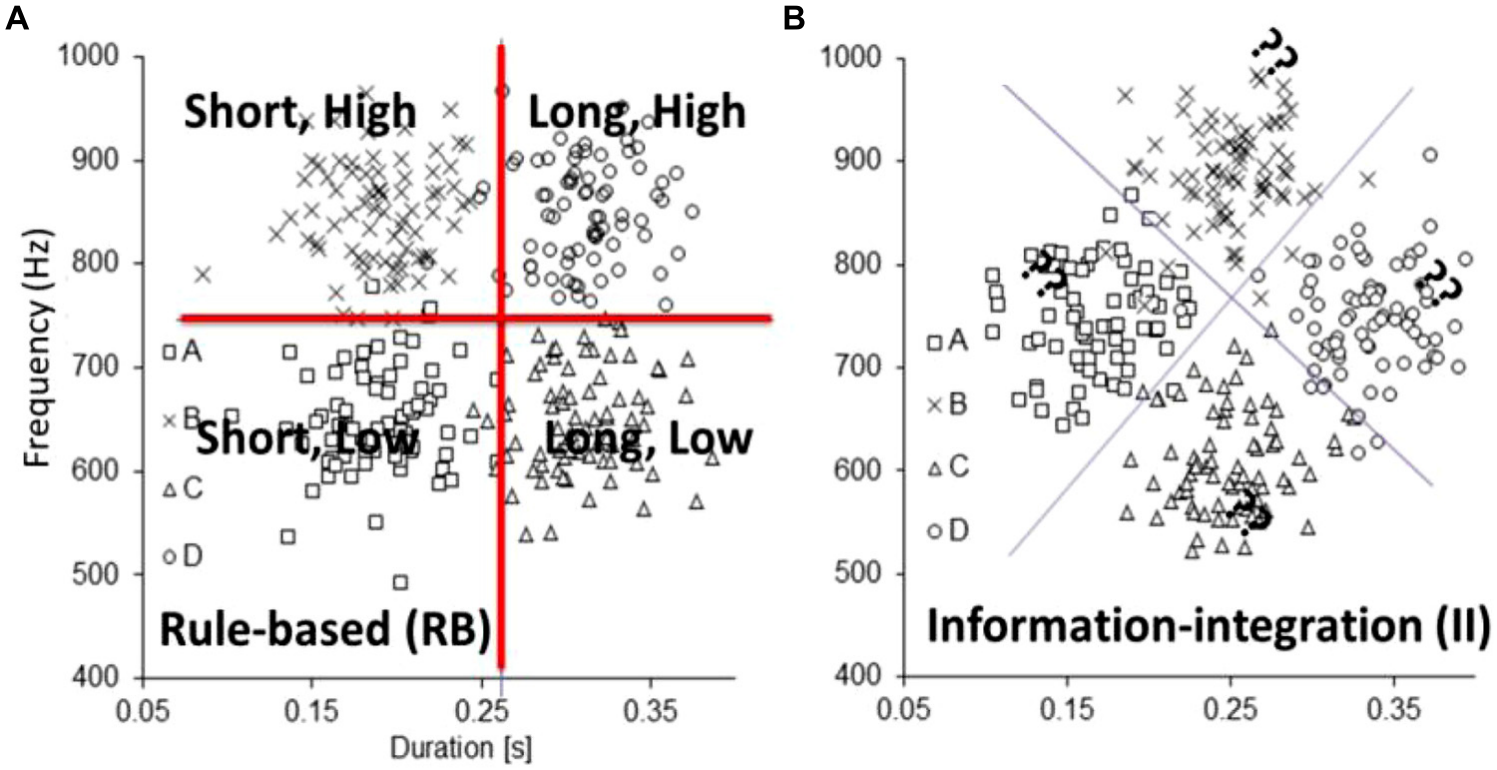
**Artificial category structures: (A) rule-based, reflective-optimal and (B) information-integration, reflexive-optimal used to study dissociations between reflective and reflexive auditory category learning systems**.

Now that we have established that the neurobiology is in place to support a dual-learning systems approach to auditory category learning, we review the empirical evidence in support of dual-learning systems using auditory category learning tasks. The most rigorous tests of dual-learning systems require the use of artificial categories for which the experimenter controls the optimal strategy and constructs one reflective-optimal and one reflexive-optimal task. **Figure 3A** displays a highly verbalizable reflective-optimal category learning problem that uses tones that vary in duration and frequency as stimuli: short, low-frequency tones are in category A; short, high-frequency tones are in category B; long, low-frequency tones are in category C; and long, high-frequency tones are in category D. In our pilot experiments, learners were able to easily verbalize their strategies for the four categories. The broken lines denote the decision boundaries that maximize accuracy.

**Figure 3B** displays a reflexive-optimal category learning problem that is constructed by rotating the **Figure 3A** stimulus space by 45°. The broken lines denote the decision boundaries that maximize accuracy. In this case, no simple verbal description exists to describe this strategy. As a proof of concept, we examined reflective-optimal and reflexive-optimal category learning in the visual domain and compared it with reflective-optimal and reflexive-optimal category learning in the auditory domain. Importantly, the category structures remained the same across the visual and auditory applications; only the specific dimensions changed. Participants showed similar learning profiles across the visual and auditory versions of the reflective-optimal and reflexive-optimal tasks, suggesting that similar mechanisms were in place. As a more rigorous test of the dual-learning systems approach, we examined whether individual differences in working memory capacity were predictive of individual differences in reflective-optimal and reflexive-optimal non-speech auditory category learning. Two lines of work in the visual domain suggest that this should matter. First, a number of researchers (Waldron and Ashby, 2001; Maddox et al., 2004; Zeithamova and Maddox, 2006, 2007; Filoteo et al., 2010) have shown that reflective-optimal visual category learning was impaired when participants were asked to perform a demanding working-memory dual task, whereas reflexive-optimal visual category learning was not affected. Second, and more directly (DeCaro et al., 2008; Tharp and Pickering, 2009; however, see Lewandowsky et al., 2012) showed that increases in working memory capacity were associated with enhanced reflective-optimal visual category learning but did not lead to advantages in reflexive-optimal visual category learning.

**FIGURE 4:**
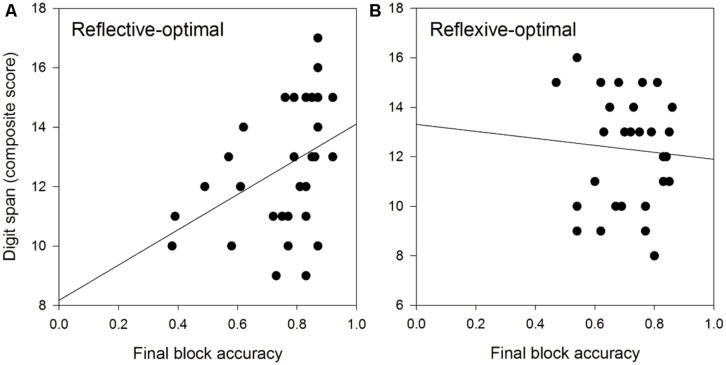
**(A)** Rule-based, reflective-optimal auditory category learning is positively related to working memory span. **(B)** Information-integration, reflexive-optimal auditory category learning is not significantly related to working memory span.

We tested this latter result directly in non-speech auditory reflective-optimal and reflexive-optimal category learning. Again, the hypothesis was that working memory would be significantly related to reflective but not reflexive processing. We had 28 young adults (18–35 years) complete the **Figure 3A** reflective-optimal non-speech auditory category learning task, and 30 young adults (18–35 years) complete **Figure 3B** reflexive-optimal non-speech auditory category learning task. Working memory capacity was assessed using the digit span portion of the Wechsler Adult Intelligence Scale, 4th edition (WAIS-IV; Wechsler, 2008). In the backward span task, numbers were read at a rate of one number per second with a monotone voice to avoid highlighting any one part of the string of numbers. Participants were required to repeat the string of numbers presented to them backwards and were scored on the sum of correct strings correctly repeated. In the forward span task, participants were required to repeat strings of numbers presented to them and were scored on the sum of strings correctly repeated. A composite span was created by adding the forward and backward spans for each participant. Figures 4A,B display scatterplots of the working memory capacity and reflective-optimal (**Figure 4A**) or reflexive-optimal (**Figure 4B**) scores.

The solid line denotes the best fitting line. As predicted, working memory capacity was significantly positively related to reflective-optimal performance, as indexed by performance on the final block (*r* = 0.393, *p* = 0.028), but was not significantly related to reflexive-optimal performance (*r* = -0.069, *p* > 0.05). This is consistent with COVIS prediction that working memory capacity is critical for learning reflective-optimal category structures, but not for learning reflexive-optimal category structures (Maddox and Ashby, 2004; Ashby and Maddox, 2005, 2010). In the next section, we review recent studies applying the COVIS model to speech category learning

### REFLECTIVE AND REFLEXIVE AUDITORY SYSTEMS IN SPEECH LEARNING

One advantage of extending COVIS to the auditory domain is that it allows the exploration of natural category learning problems. Speech perception can be likened to a categorization problem, in which, multidimensional and highly variable acoustic signals are needed to be parsed into discrete phonological representations. One exciting possibility is that dual-learning systems may underlie speech category learning, which is one of the most difficult human category learning problems. The ability to learn and understand (categorize) speech sounds, either as a first or second language, is a critical skill at which humans are remarkably adept. In fact, as anyone who has experience with the speech recognition systems associated with many “smart” phones knows, the human ability to understand speech far out weights that of even the most sophisticated computer algorithm. The multidimensional and highly variable characteristics of speech signals make speech learning a “difficult” categorization problem, especially for individuals learning novel speech categories in adulthood.

Previous research has theorized several reasons for difficulties in the acquisition of second language (L2) speech categories. These difficulties have been interference caused by existing speech categories, as well as interference due to a “warping” of auditory-perceptual space by prior experience with native speech categories (Flege, 1999; Francis and Nusbaum, 2002; Kilpatrick et al., 2003; Francis et al., 2008). Although difficult, adults can acquire L2 speech categories. Laboratory training paradigms ubiquitously utilize trial-by-trial feedback and high-variability (multiple speakers) training to teach L2 speech categories (Lively et al., 1993; Bradlow et al., 1999; Tricomi et al., 2006; Zhang et al., 2009; Lim and Holt, 2011). Feedback is thought to enhance learning by reducing errors, and multiple-speaker training results in learners refocusing their attention to cues that are relevant for distinguishing speech categories and/or reducing attention to irrelevant cues (Bradlow and Bent, 2008). Although unsupervised training results in some amount of speech learning in adults, the addition of feedback results in substantially larger learning gains (McClelland et al., 2002; Vallabha and McClelland, 2007; Goudbeek et al., 2008). Studies have also examined the role of high-variability (multiple-speaker) training in speech learning. While much of this research has focused on the mechanics of the perceptual system in speech learning, much less is known about the role of the dual-learning systems, which previous studies suggest is critical to learning reflective-optimal and reflexive-optimal category structures. This leads us to an important question: are speech categories similar to reflective-optimal category structures or reflexive-optimal category structures?

Speech categories typically are difficult to verbalize, have multiple dimensions, and are highly variable. Generating and testing hypotheses for categories involving multiple dimensions is resource-intensive. Since the reflective system is dependent on working memory and attention, generating rules/hypotheses for multiple dimensions may not be efficient. Furthermore, the redundancy and variability of cues available during speech perception prevents a simple one-to-one mapping of cues to categories. These suggest that reflexive learning may be most optimal for speech categories. Our hypothesis is therefore that speech learning is reflexive-optimal. During natural visual category learning, the dual-learning systems framework assumes that the reflective and reflexive learning systems compete throughout learning for control (Ashby and Maddox, 2011). Early in category learning, the dual-learning systems model assumes that learners are mostly reflective. They actively test a number of hypotheses and use feedback to validate or invalidate rules. With practice, learners switch to the more automatic, reflexive learning system if the output of this system is more accurate than the reflective system. In line with dual-learning systems predictions, we propose that learning speech category structures is reflexive-optimal and that successful learners may initially use reflective strategies but eventually switch to the more optimal (reflexive) learning system. We have conducted a series of experiments to test this hypothesis. In the next section, we will briefly discuss the major points from these studies.

## APPLICATION 1: IS SPEECH LEARNING REFLECTIVE- OR REFLEXIVE-OPTIMAL? CHANDRASEKARAN ET AL. (2014)

As outlined above, our working hypothesis is that speech categories are optimally learned by the *reflexive* learning system (Chandrasekaran et al., 2014). This is because speech categories are often difficult to verbalize and utilize acoustic cues that are multidimensional, highly redundant, and variable across speakers (Gandour, 1983; Holt and Lotto, 2008, 2010). Creating rules for such complex category structures may not be optimal, since generating and testing rules that involve multiple dimensions is resource intensive. Chandrasekaran et al. (2014) utilized the dissociation logic developed to test COVIS and training manipulations on trial-by-trial feedback (Experiments 1 and 2) and speaker variability (Experiment 3) to examine the relative contribution of the reflective and reflexive learning systems to speech learning success. The reflective and reflexive learning systems have been shown to respond differentially to various training manipulations. For example, delaying the presentation of feedback impairs learning in the reflexive system, but not in the reflective system (Maddox et al., 2003; Maddox and Ing, 2005). This is because the reflexive system is critically dependent on dopamine-mediated stimulus-response implicit reward learning. Delaying feedback interferes with dopamine release, reducing the effectiveness of the association of stimulus-response with reward. Also, rich, informational, “full” feedback that provides the correctness of the response on each trial as well as information about which category was present speeds learning in the reflective system (Maddox et al., 2008) relative to “minimal” feedback that provides only the correctness of the response on each trial. Full feedback promotes the generation and testing of rules that are critical to reflective learning but disrupts the transfer of control to the reflexive system (Maddox et al., 2008). Previous studies have used these timing and feedback manipulations to dissociate the learning systems in artificial visual category learning, but not in natural speech category learning.

**FIGURE 5:**
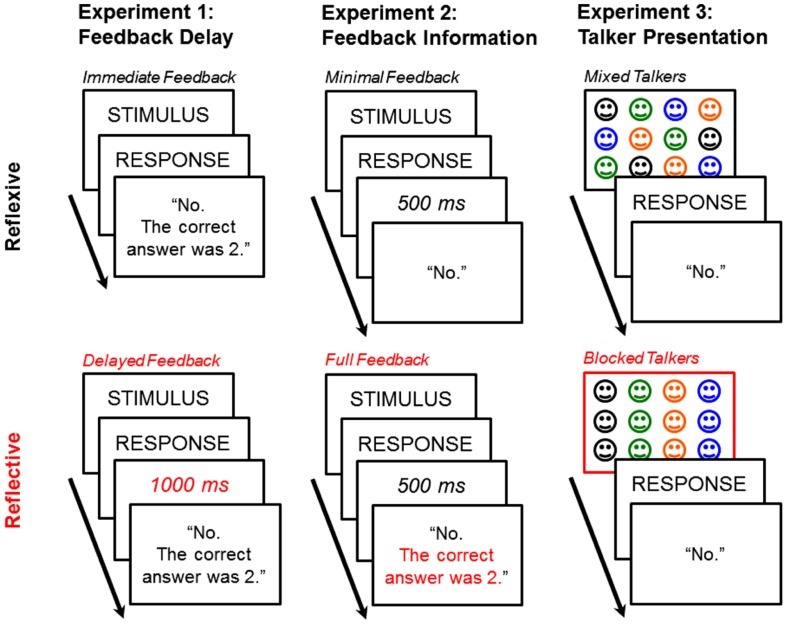
**Experimental procedures from Chandrasekaran et al. (2014).** In Experiments 1–3, we examined the effects of reflexive (top panel) or reflective (bottom panel) training manipulations on tone category learning success.

Experiment 1 determined the extent to which the *immediacy* of feedback (immediate vs. delayed) impacts tone category learning. Experiment 2 determined the extent to which the *information content* of feedback (full versus minimal feedback) impacts tone category learning (**Figure 5**). Immediate feedback is critical for the reflexive system but not the reflective system (Maddox et al., 2003), while full feedback selectively speeds reflective learning but impairs reflexive learning (Maddox et al., 2008). Based on our working hypothesis, we predicted that feedback manipulations that targeted the reflexive learning system (immediate or minimal feedback) would enhance learning relative to those that target the reflective learning system (delayed or full feedback).

While dual-learning systems models of visual category learning make specific predictions about feedback processing, they offer no clear prediction about the impact of speaker variability on category learning success. While multi-speaker training is argued to be advantageous in generalizing to speech produced by novel speakers, the role of the order of speaker presentation, if any, has not been systematically examined in previous research. Within the framework of the dual-learning systems, we predicted that systematically blocked speaker presentation (i.e., presenting all stimuli from one speaker) will promote reflective learning, whereas a randomly mixed-speaker presentation will enhance reflexive learning. Our logic here is that blocked speaker presentation promotes faster hypothesis testing and validation, and is therefore less resource intensive for the reflective system than is the mixed-speaker condition. Also, the mixed-speaker presentation does not allow learners to predict the next speaker in advance, disrupting the immediate testing of speaker-specific rules. Therefore, our prediction is that learners are more likely to associate speaker-invariant acoustic cues with implicit reward than speaker-variant cues. Based on the hypothesis that speech learning is optimally learned by the reflexive learning system, we predicted enhanced learning in the mixed-speaker condition, relative to the blocked speaker condition.

### SPEECH CATEGORY LEARNING TASK

**FIGURE 6:**
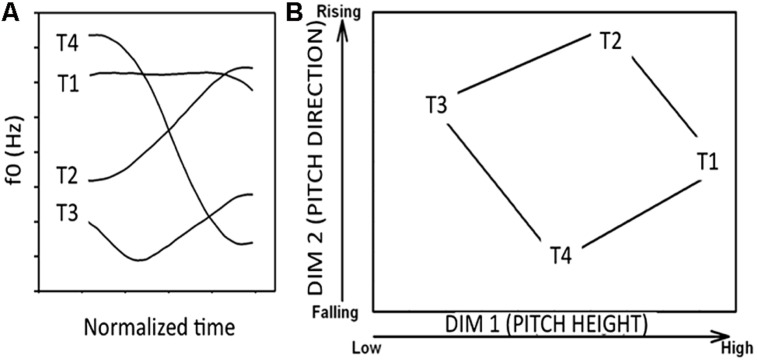
**(A)** Sample fundamental frequency contours of four Mandarin tones (T1: high-level; T2: low-rising; T3: low-dipping; T4: high-falling) produced by a male native Mandarin speaker. **(B)** The four tones plotted in a two-dimensional perceptual space (*x*-axis: pitch height, *y*-axis: pitch direction). Pitch height (dimension 1) and pitch direction (dimension 2) are major cues used to distinguish the tone categories.

To study L2 speech category learning, we utilized naturally produced Mandarin tone categories, which are non-native to monolingual English speakers. Mandarin Chinese has four tone categories [ma^1^ “mother” [T1], ma^2^ “hemp” [T2], ma^3^^“^horse” [T3], ma^4^ “scold” [T4]), described phonetically as high level, low rising, low dipping, and high falling, respectively (**Figure 6A**). Native English speakers find it particularly difficult to learn tone categories (Wang et al., 2003). However, previous studies also show that short-term laboratory training can enhance tone identification and discrimination in native English speakers, although such training paradigms have typically resulted in significant inter-individual differences in learning success (Perrachione et al., 2011).

A number of dimensions (e.g., pitch height, pitch direction) may serve as cues to tone categorization. The relative perceptual saliency of these dimensions is influenced by the presence or absence of pitch patterns in a language’s tonal inventory (Gandour, 1978, 1983) as well as by the occurrence of abstract rules in a listeners’ phonological system (Hume and Johnson, 2001). Multidimensional scaling studies on tone perception converge on two primary dimensions that underlie the tone space: labeled pitch height and pitch direction (**Figure 6**).

**FIGURE 7:**
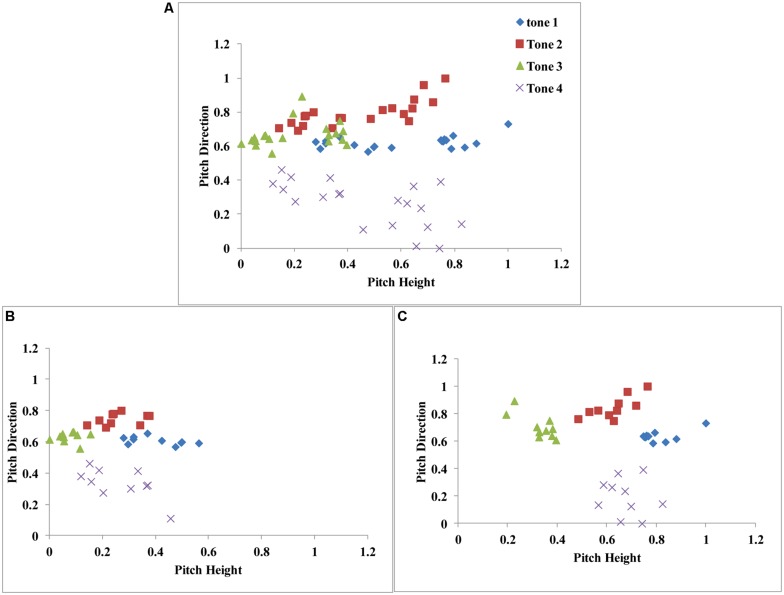
**(A)** Scatterplot of all stimuli from the Mandarin tone category learning experiment. **(B)** Scatterplot of male-speaker stimuli. **(C)** Scatterplot of female-speaker stimuli. Stimulus dimensions (pitch height and pitch direction) were normalized between 0 and 1.

In **Figure 7A**, we plot the 80 stimuli used in our experiments (five consonant–vowel segments X four speakers X four tones) along two dimensions [pitch height: average fundamental frequency (*x*-axis) and pitch direction: slope (*y*-axis)]. A visual inspection of this space supports our hypothesis that speech category learning is reflexive-optimal (similar to the structure in **Figure 3B**). That is, category separation is greatest when the dimensions (pitch height and direction) are integrated in a manner that is not easily verbalizable.

### RESULTS FROM CHANDRASEKARAN ET AL. (2014)

**FIGURE 8:**
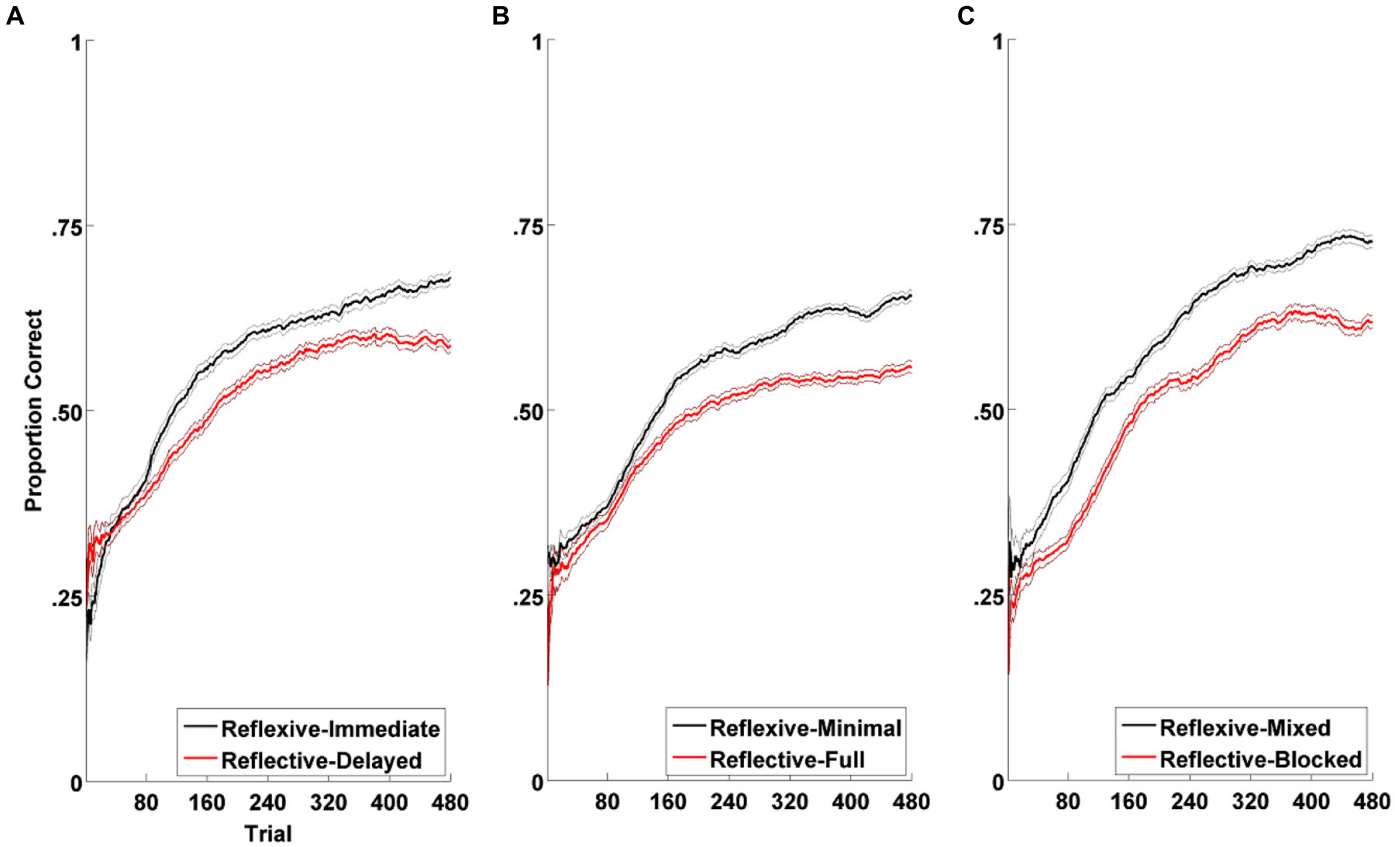
**Category learning curves across reflexive vs. reflective conditions in all three experiments from Chandrasekaran et al. (2014): (A) Experiment 1: feedback delay (immediate vs. delayed); (B) Experiment 2: feedback information (minimal vs. full); (C) Experiment 3: speaker variability (mixed vs. blocked).** Plotted in solid bold lines are the proportions of correct responses across participants within each condition over the course of learning. The black lines denote the reflexive conditions and the red, the reflective conditions. For purposes of visualization of trial-by-trial data, each point in the line denotes the average number of correct responses in a sliding 80-trial window. For trials preceding the 80th trial, cumulative averages were used. Plotted in thin lines are the ranges of standard error of the averages used in the sliding windows. Visual assessment of the learning curves suggest that both conditions result in equivalent degrees of category learning toward the earlier phase of experiment, but that the reflexive condition leads to greater learning than does the reflective condition toward the later phase of the experiment. This pattern is consistent across all three experiments.

**Figure 8** summarizes the results from the three experiments. In all cases, the training manipulation hypothesized to enhance reflexive learning led to better long-term Mandarin tone learning than the training manipulation hypothesized to enhance reflective learning. Taken together, these data provide strong support for the prediction that natural speech category learning is reflexive-optimal.

## APPLICATION 2: COMPUTATIONAL MODELS AS A WINDOW ONTO COGNITIVE PROCESSING: A REANALYSIS OF CHANDRASEKARAN ET AL. (2014)

Chandrasekaran et al. (2014) relied on behavioral measures of accuracy to determine whether L2 speech category learning was reflective-optimal or reflexive-optimal. Although a good starting point, one weakness of accuracy-based measures is that the same accuracy rate can often be achieved by using qualitatively different strategies (e.g., reflective or reflexive). Within the domain of category learning, computational models can be utilized that address this shortcoming and can provide important insights into the nature of the strategy (reflective/reflexive) that an individual is applying in a given task. We predict that individuals in the immediate feedback, minimal feedback, and mixed-speaker conditions will utilize reflexive strategies to a greater degree than individuals in the delayed feedback, rich informational feedback, and blocked speaker conditions.

To test this hypothesis, we applied a series of decision-bound models developed by Maddox and Chandrasekaran (in press) on a block-by-block basis at the individual participant level. This was due to problems with interpreting fits to aggregate data (Estes, 1956; Ashby et al., 1994; Maddox, 1999). We assume that the two-dimensional space (pitch height vs. pitch direction) displayed in **Figure 7A** accurately describes the perceptual representation of the stimuli. Based on the results from our earlier work (Maddox and Chandrasekaran, in press), we also assumed that participants applied category learning strategies separately to the male (**Figure 7B**) and female (**Figure 7C**) perceptual spaces. Note that, as long as the major dimensions are known, these modeling procedures can be applied to any type of speech category structure. This offers an exciting new approach to the study of speech categorization.

### MODEL DETAILS

Here we provide a brief description of each model. More details are available in numerous previous publications (e.g., Ashby and Maddox, 1993; Maddox and Ashby, 1993; Maddox and Chandrasekaran, in press). Each model assumes that decision bounds were used to classify stimuli into each of the four Mandarin tone categories (T1, T2, T3, or T4). The model-based approach involves applying three classes of models, with multiple instantiations possible within a class. The first class is computational models of the reflexive procedural learning system. This is instantiated with the Striatal Pattern Classifier (SPC; Ashby and Waldron, 1999; Maddox et al., 2002b). The SPC is a computational model whose processing is consistent with what is known about the neurobiology of the procedural-based category learning system thought to underlie II classification performance (Ashby et al., 1998; Maddox et al., 2002a; Seger and Cincotta, 2005; Ashby and Ennis, 2006; Nomura et al., 2007). The second class is reflective, RB and instantiate hypothesis-testing strategies, such as the application of unidimensional or conjunctive rules. These are verbalizable strategies. The third model is a random responder model that assumes that the participant guesses on each trial. The model parameters were estimated using maximum likelihood procedures (Wickens, 1982; Ashby, 1992) and models were compared using Akaike weights (Wagenmakers and Farrell, 2004). These detailed analyses are available in the original manuscript. We provide the specifics of each model in the next section.

#### Striatal pattern classifiers

**FIGURE 9:**
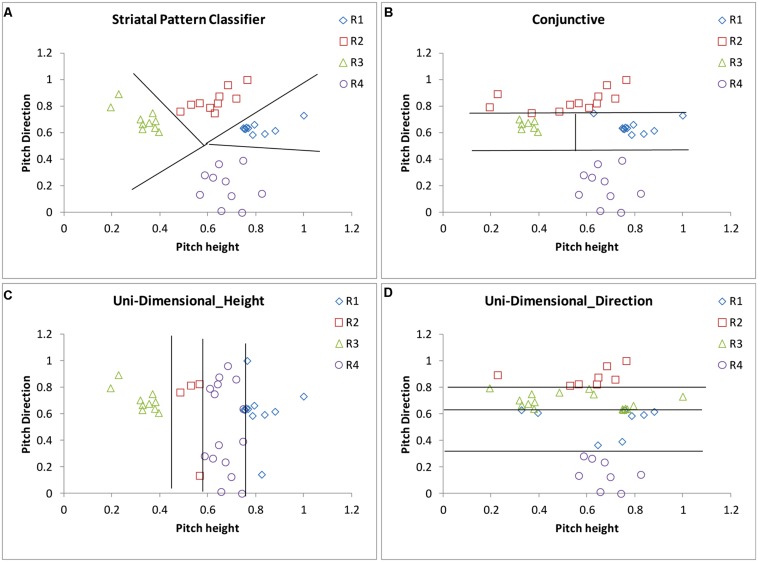
**Scatterplots of the responses along with the decision boundaries that separate response regions from a hypothetical participant using a version of the (A) Striatal Pattern Classifier, (B) Conjunctive rule-based, (C) Uni-Dimensional Height, and (D) Uni-Dimensional Direction models as applied to the female-speaker stimuli shown in **Figure 7C****.

The SPC assumes that stimuli are represented perceptually in higher level auditory areas, such as the superior temporal gyrus. Because of the massive many-to-one (approximately 10,000-to-1) convergence of afferents from the primary and secondary sensory cortices to the striatum (Wilson, 1995; Ashby and Ennis, 2006), a low-resolution map of perceptual space is represented among the *striatal units*. Within the auditory domain, it is well known that there are direct projections from secondary auditory areas such as superior temporal gyrus and supratemporal plane to the caudate (Hikosaka et al., 1989; Arnauld et al., 1996; Yeterian and Pandya, 1998). During feedback-based learning, the striatal units become associated with one of the category labels so that, after learning is complete, a category response label is associated with each of a number of different regions of perceptual space. In effect, the striatum learns to associate a response with clumps of cells in the auditory cortex. It is important to be clear that the SPC is a computational model that is inspired by what is known about the neurobiology of the striatum. Because of this fact, the striatal “units” are hypothetical and could be interpreted within the language of other computational models (e.g., as “prototypes” in a multiple prototype model like SUSTAIN; Love et al., 2004). In addition, we do not model learning in the SPC in the sense that we do not update association weights between units and category labels. Learning models have been proposed (Ashby and Maddox, 2011) but are not utilized here due to their complexity. The SPC assumes that there is one striatal “unit” in the pitch height–pitch direction space for each category, and a single “noise” parameter that represents the noise associated with the placement of the striatal units. Responses from a hypothetical participant using the SPC are displayed in **Figure 9A**.

#### Conjunctive rule-based model

A conjunctive RB model that assumes that the participant sets two criteria along the pitch direction dimension and one criterion along the pitch height dimension was also applied to the data. The model assumes that the two criteria along the pitch direction dimension are used to separate the stimuli into those that are of low, medium, or high pitch direction. Low pitch direction items are classified into tone category 4 (T4) and high pitch direction items are classified into tone category 2 (T2). If an item is classified as having medium pitch direction, then the pitch height dimension is examined. The single criterion along the pitch height dimension is used to separate the stimuli into low and high pitch height. Stimuli that have medium pitch direction and low pitch height are classified into tone category 3 (T3) and medium pitch direction items of high pitch height are classified into tone category 1 (T1). Responses from a hypothetical participant using a conjunctive strategy are displayed in **Figure 9B**.

#### Unidimensional rule-based model

A unidimensional height RB model that assumes that the participant sets three criteria along the pitch height dimension was also applied to the data. The model assumes that the three criteria along the pitch height dimension are used to separate the stimuli into those that are of low, medium–low, medium–high or high pitch height, with each of these being associated with one of the four tone categories. Notice that this model completely ignores the pitch direction dimension. Although 24 versions of the model are possible given four category labels, some are highly unrealistic [e.g., a model that assumes that tone category 1 (T1) was the lowest in pitch height]. We examined the eight most reasonable variants of the model.

A unidimensional direction RB model that assumes that the participant sets three criteria along the pitch direction dimension was also applied to the data. The model assumes that the three criteria along the pitch direction dimension are used to separate the stimuli into those that are of low, medium-low, medium-high, or high pitch direction with each of these being associated with one of the tone categories. Notice that this model completely ignores the pitch height dimension. Although 24 versions of the model are possible given four category labels, many are highly unrealistic. We examined the two most reasonable variants of the model. Responses from a hypothetical participant using a unidimensional strategy along pitch height are displayed in **Figure 9C**, and responses from a hypothetical participant using a uni-dimensional strategy along pitch direction are displayed in **Figure 9D**.

#### Random responder model

The random responder model assumes a fixed probability of responding tone 1, tone 2, tone 3, and tone 4 but allows for response biases. The model has three free parameters to denote the predicted probability of responding “1,” “2,” or “3” with the probability of responding “4” equal to one minus the sum for the other three categories.

### MODEL RESULTS

As outlined in Application 1, we found better learning when feedback was immediate relative to delayed, when feedback was minimal relative to informationally rich, and when speaker presentation was mixed as opposed to blocked. We assumed that these performance advantages were due to better utilization of the reflexive system. As a test of this hypothesis, we fit the models outlined above to the data from the published study, focusing on the final block. In line with our predictions, we found that 53% of participant’s final block data in the immediate feedback condition was best fit by the SPC, whereas only 43% of participant’s final block data in the delayed feedback condition was best fit by the SPC. Analogously, we found that 53% of participant’s final block data in the minimal feedback condition was best fit by the SPC whereas only 42% of participant’s final block data in the informationally rich feedback condition was best fit by the SPC. Finally, and again in support of our hypothesis, we found that 67% of participant’s final block data in the mixed-speaker condition was best fit by the SPC whereas only 50% of participant’s final block data in the blocked speaker condition was best fit by the SPC.

## APPLICATION 3: INDIVIDUAL DIFFERENCES IN SPEECH CATEGORY LEARNING

### SPEECH CATEGORY LEARNING ACROSS THE LIFESPAN

One of our first applications of the dual-learning systems approach in the auditory domain was to examine the effect of normal aging on category learning. Little is known about the learning systems that mediate successful auditory and speech categorization across the lifespan. Normal aging is associated with some deficiencies in reflective and reflexive category learning within the visual domain (Ashby et al., 2003; Maddox et al., 2010), but these have not been explored in the auditory domain. Particularly, previous studies have demonstrated age-related declines in working memory and prefrontal function that may disproportionally impact learning reflective category structures (Daigneault and Braun, 1993; West, 1996; Clapp et al., 2011). We used experimental and computational modeling approaches to examine the extent to which dual-learning systems mediate speech learning in younger and older adults (Maddox et al., 2013). We used the same task outlined in Applications 1 and 2. We did have to make a minor change to get reasonable learning within a single session, and that was to include only one male and one female speaker instead of two male and two female speakers. This change led to only small differences in predicted accuracy across the reflective-conjunctive model and the reflexive-SPC model. However, reflective unidimensional models predicted poor accuracy.

**FIGURE 10:**
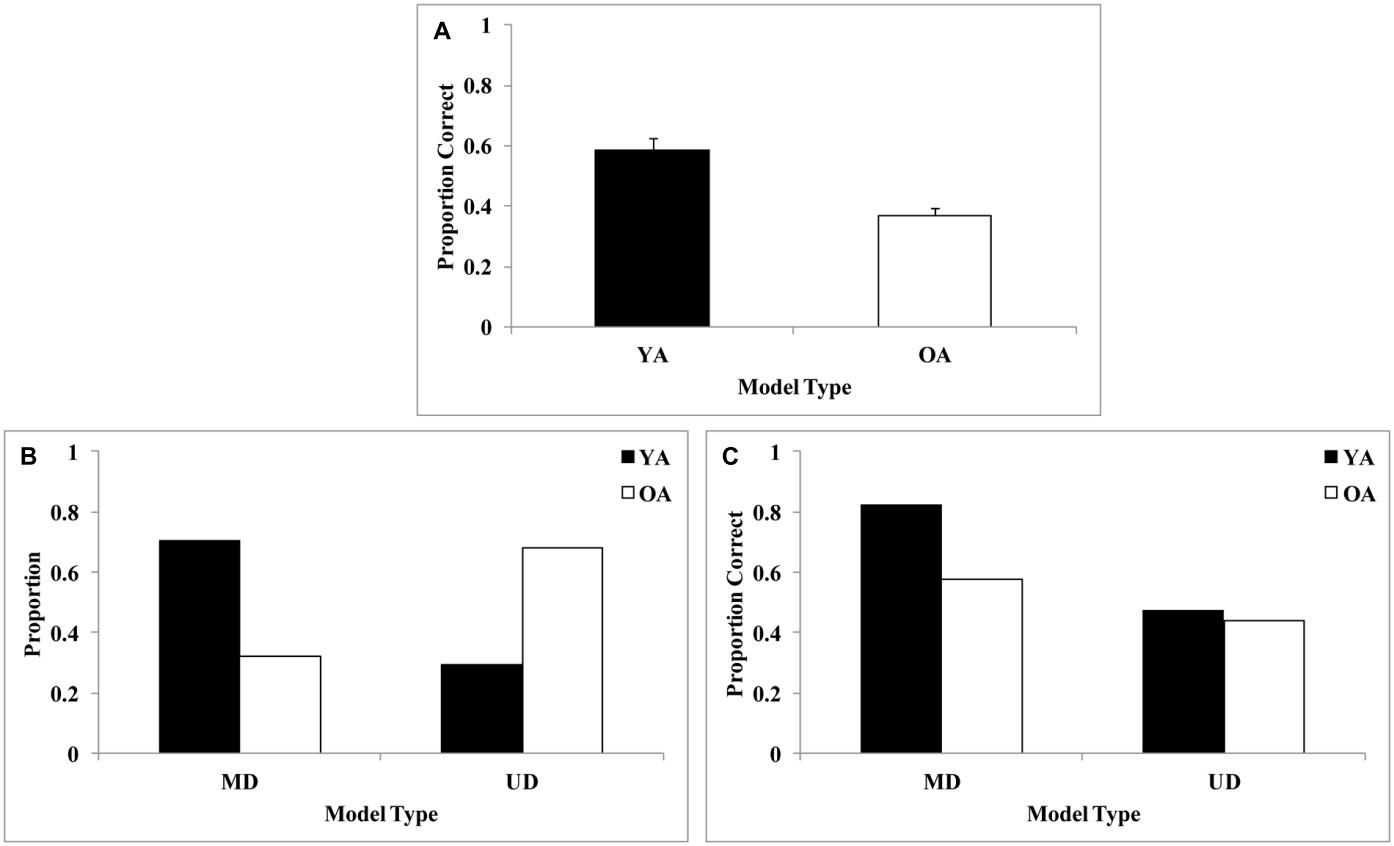
**(A)** Overall accuracy across older adults (OA) and younger adults (YA), **(B)** final block proportion of multi-dimensional [Striatal Pattern Classifier (SPC)/conjunctive rule-based (CJ)] and uni-dimensional (UD) models, and **(C)** final block accuracy for each model type by age group from Maddox et al. (2013). In this particular experiment’s stimulus set, SPC and CJ model fits were effectively inseparable, and so have been collapsed in this analysis. Older adults use a greater proportion of simple unidimensional rules, likely due to a deficit in the reflective learning system.

We found an age-related deficit in overall performance that is displayed in **Figure 10A**. **Figure 10B** displays the proportion of older and younger adults whose final block of data was best fit by a multi-dimensional model (conjunctive or SPC) or a unidimensional model. Whereas approximately 70% of younger adults were using a multi-dimensional model, only about 30% of older adults were using a multi-dimensional model. Thus, older adults generally perseverated on unidimensional rules when the optimal strategy was to focus on both dimensions. The perseveration on simple unidimensional rules is likely due to a deficit in the reflective learning system. However, due to the fact that we could not separate conjunctive and SPC models, we cannot make a definite conclusion regarding a reflective learning deficit in older adults. This result mirrored previous results in the visual domain, where older adults were slower to transition from RB to procedural rules (Maddox et al., 2010). Next, we examined the final block accuracy rates for older and younger adults as a function of strategy type (**Figure 10C**). Interestingly, younger adults who used multi-dimensional strategies were more accurate than older adults who used multi-dimensional strategies. However, older and younger adults who used unidimensional strategies yielded about the same (low) accuracy rates. Taken together, these data suggest that younger adults are more likely than older adults to shift from suboptimal uni-dimensional to optimal multi-dimensional strategies, and even when older adults do shift to optimal multi-dimensional strategies, they use these less accurately than younger adults.

### INFLUENCE OF DEPRESSIVE SYMPTOMS ON SPEECH CATEGORY LEARNING

A second application of the dual-learning systems approach in the auditory domain was to examine the effect of elevated depressive symptoms on category learning (Maddox et al., 2014). Little is known about the learning systems that mediate successful auditory and speech categorization in individuals with elevated depressive symptoms. Previous studies have shown that individuals with elevated depressive symptoms show deficits in reflective processing (Beevers, 2005; Carver et al., 2009; Beevers et al., 2012; Maddox et al., 2012; Blanco et al., 2013), and because of the deficit in frontally mediated processes, like working memory and cognitive flexibility, we would predict impaired performance on auditory reflective-optimal tasks. We exploited this finding to test critical predictions of the dual-learning systems model in audition. Because the reflective and reflexive systems are dissociable and competitive, we predicted that elevated depressive symptoms would lead to reflective-optimal learning deficits but reflexive-optimal learning *advantages*. Because natural speech category learning is reflexive in nature, we made the prediction that elevated depressive symptoms would lead to *superior* speech learning. In support of our predictions, individuals with elevated depressive symptoms showed a deficit in reflective-optimal auditory category learning, but an advantage in reflexive-optimal auditory category learning. In addition, using the same stimuli in **Figure 7**, we found that individuals with elevated depressive symptoms showed an advantage in learning a non-native speech category structure. Computational modeling suggested that the elevated depressive symptom advantage was due to faster, more accurate, and more frequent use of reflexive category learning strategies in individuals with elevated depressive symptoms.

## SUMMARY AND FUTURE DIRECTIONS

Auditory category learning has been traditionally viewed as a perceptually encapsulated process. In contrast, the dual-learning systems theoretical approach tackles learning from an auditory-cognitive categorization perspective. This is an important step toward assessing domain-general influences on auditory and speech processing. Popular dual-learning systems models in vision have been cautious about extending this model beyond vision because the neurobiological plausibility of dual-learning systems in audition has not been extensively studied. Here we argue that the reflective and reflexive learning systems are neurobiologically viable in audition. Moreover, behavioral and computational modeling work clearly demonstrates a functional role for these systems in learning a variety of auditory categories. From a practical standpoint, understanding the role of the dual-learning systems may inform language pedagogy. Extant auditory training programs for language and music pedagogy may be suboptimal because the dynamics of feedback provided are arbitrary and do not target the learning system that is optimal for learning a particular auditory category structure. Our experiments clearly establish the optimal set of feedback characteristics for a broad range of auditory category problems. These training procedures can be easily incorporated into existing auditory training programs and language software, and may have a significant theoretical and practical impact on language and music pedagogy.

## Conflict of Interest Statement

The authors declare that the research was conducted in the absence of any commercial or financial relationships that could be construed as a potential conflict of interest.

## Footnotes

1 Recent evidence suggests a third system, referred to as the perceptual-representation system, can also mediate category learning under certain conditions (Casale and Ashby, 2008; Zeithamova et al., 2008).
